# Overexpression of CD97 in Intestinal Epithelial Cells of Transgenic Mice Attenuates Colitis by Strengthening Adherens Junctions

**DOI:** 10.1371/journal.pone.0008507

**Published:** 2010-01-13

**Authors:** Susann Becker, Elke Wandel, Manja Wobus, Rick Schneider, Salah Amasheh, Doreen Sittig, Christiane Kerner, Ronald Naumann, Joerg Hamann, Gabriela Aust

**Affiliations:** 1 Research Laboratories, Department of Surgery, University of Leipzig, Leipzig, Germany; 2 Translational Centre for Regenerative Medicine, University of Leipzig, Leipzig, Germany; 3 Institute of Clinical Physiology, Charité, Berlin, Germany; 4 Transgenic Core Facility, MPI for Molecular Cell Biology and Genetics, Dresden, Germany; 5 Department of Experimental Immunology, Academic Medical Center, University of Amsterdam, Amsterdam, The Netherlands; Charité-Universitätsmedizin Berlin, Germany

## Abstract

The adhesion G-protein-coupled receptor CD97 is present in normal colonic enterocytes but overexpressed in colorectal carcinoma. To investigate the function of CD97 in colorectal carcinogenesis, transgenic Tg(villin-CD97) mice overexpressing CD97 in enterocytes were generated and subjected to azoxymethane (AOM)/dextran sodium sulfate (DSS)-induced colitis-associated tumorigenesis. Unexpectedly, we found a CD97 cDNA copy number-dependent reduction of DSS-induced colitis in Tg compared to wild-type (WT) mice that was confirmed by applying a simple DSS protocol. Ultrastructural analysis revealed that overexpression of CD97 strengthened lateral cell-cell contacts between enterocytes, which, in contrast, were weakened in CD97 knockout (Ko) mice. Transepithelial resistance was not altered in Tg and Ko mice, indicating that tight junctions were not affected. In Tg murine and normal human colonic enterocytes as well as in colorectal cell lines CD97 was localized preferentially in E-cadherin-based adherens junctions. CD97 overexpression upregulated membrane-bound but not cytoplasmic or nuclear β-catenin and reduced phospho-β-catenin, labeled for degradation. This was associated with inactivation of glycogen synthase kinase-3β (GSK-3β) and activation of Akt. In summary, CD97 increases the structural integrity of enterocytic adherens junctions by increasing and stabilizing junctional β-catenin, thereby regulating intestinal epithelial strength and attenuating experimental colitis.

## Introduction

Intestinal epithelial cells are linked together by a junctional complex comprising tight and adherens junctions and desmosomes, providing different functions in cell-cell adhesion. In adherens junctions (or *zonulae adherentes*), homophilically interacting E-cadherin receptors physically link the confronting cell membranes. At the intracellular membrane face, E-cadherins are connected through intracellular plaque core proteins, mainly catenins, to cortical actin filament bundles, organizing a circular adhesion belt [1], [2]. E-cadherin-based adherens junctions contribute crucially to the organization and stabilization of the polarized intestinal cell layer. Their dysregulation causes cell depolarization, loss of contact-dependent inhibition of proliferation, increased motility, leakiness of the mucosal barrier, and disturbances in enterocytic cell-cell contact formation – processes that have been linked to colorectal carcinogenesis [3] and inflammatory bowel disease [4]–[6]. Additional complications can arise from the dislocation of membrane-bound β-catenin, which functions as a nuclear modulator in Wnt signaling during colorectal carcinogenesis [2].

An increasing number of molecules have been described to be engaged in the complex regulation of adherens junctions, a process which is not yet understood in detail [7], [8]. Here, we report CD97 to be located in enterocytic E-cadherin-based adherens junctions, where it regulates structural integrity by modulating the anchor protein β-catenin. CD97 is a member of the epidermal growth factor seven-span transmembrane (EGF-TM7) family of adhesion G-protein-coupled receptors [9]. Next to its broad and abundant expression in leukocytes, CD97 is found in various normal and malignant epithelia. We previously described its overexpression in colorectal adenocarcinomas compared to the corresponding normal tissues [10]. Remarkably, scattered colorectal tumor cells at the tumor-host interface express significantly more CD97 than cells in solid tumor formations. Corroborating this observation, CD97 expression levels correlated with the migratory and invasive capacity of colorectal tumor cell lines *in vitro* [10]. CD97 overexpression in HT1080 human fibrosarcoma cells promoted tumor growth in *scid* mice and stimulated single cell motility *in vitro* in an isoform-specific manner [11]. *In silico* simulations suggested that CD97 can increase the invasive capacity of tumors and cause the appearance of scattered tumor cells at the invasion front [11].

To explore directly the hypothesis that CD97 expression affects colorectal carcinogenesis, we generated transgenic (Tg) mice that constitutively overexpress CD97 in intestinal epithelial cells. These CD97 Tg mice were examined in the azoxymethane (AOM)/dextran sodium sulfate (DSS) model for colitis-associated tumorigenesis. Unexpectedly, depending on the CD97 cDNA copy number integrated, carcinogenesis in Tg mice was reduced due to impaired DSS-induced injury. Ultrastructural analysis of colonic enterocytes revealed that lateral cell-cell contacts were strengthened in CD97 Tg mice and weakened in CD97 knockout (Ko) mice. We demonstrate that CD97 is located in E-cadherin-based adherens junctions and that it regulates membrane-associated β-catenin associated with alterations in Akt/glycogen synthase kinase-3β (GSK-3β) signaling.

## Materials and Methods

### Ethics Statement

This research complied with the ethics guidelines of the University of Leipzig. For the generation of transgenic mice and for animal experiments we obtained ethics approval from the Landesdirektion Leipzig (TVV01/06, TVV23/08). We obtained ethics approval from the Ethics Committee of the Medical Faculty of the University of Leipzig (No111-2009) to analyze human colonic samples and written consent from all participants involved in this study.

### Reagents

Primers and antibodies used in this study are specified in table 1 and 2.

**Table 1:** List of primers.

Name	Sequence (5′-…-3′)	Application
mCD97-s1	ccg cgt acg gcc acc atg agg agc gtc	cloning
mCD97-r1	tgt cac gcg tgg aac tcg cct tca cat	cloning
villin-s1	tgc ctt ctc ctc tag gct cgt	amplification of the transgene, copy number determination
mCD97-r2	ggg cga tgg cgg tga tgg tc	amplification of the transgene
mCD97-r3	aaa gtc tcc aca gga aaa tcc	copy number determination
mCD97-s2	cct ggt cgg cgt gga gaa tga ag	real-time PCR mouse tissues
mCD97-r4	ggg cga tgg cgg tga tgg tc	real-time PCR mouse tissues
mGAPDH-s1	tcc acc acc ctg ttg ctg ta	real-time PCR mouse tissues
mGAPDH-r1	acc aca gtc cat gcc atc ac	real-time PCR mouse tissues

**Table 2:** List of antibodies.

Antigen	Species specificity	Origin	Clone	Company/reference
CD97, α-chain	mouse	hamster monoclonal	1B2	[13]
CD97, α-chain	mouse	goat polyclonal		R&D Systems, Wiesbaden, Germany; [14]
CD97, β-chain	mouse	rabbit polyclonal		[14]
CD97, α-chain	human	mouse monoclonal	CLB-CD97/3	[36]
CD97, α-chain (aa 220–270)	human	rabbit polyclonal		Imgenex, San Diego, USA
myeloperoxidase	mouse	rabbit polyclonal		GeneTex Inc., San Antonio, USA
α-catenin	mouse/human	rabbit polyclonal		GeneTex Inc
α-tubulin	mouse/human	mouse monoclonal	DM1A	Sigma-Aldrich, Taufkirchen, Germany
β-catenin	mouse/human	rabbit polyclonal		Santa Cruz
phospho-β catenin (Ser33/Ser37)	mouse/human	mouse monoclonal	BC-22	Santa Cruz
E-cadherin	mouse/human	rat monoclonal	DECMA-1	Abcam, Cambridge, UK
EpCAM	mouse	rat monoclonal	G8.8	BD Biosciences, Heidelberg, Germany
desmoplakin	mouse/human	rabbit polyclonal		Santa Cruz, Heidelberg, Germany
H3 histone	mouse/human	rabbit monoclonal	E173-58	Epitomics Inc., Burlingame, USA
p120 catenin	mouse/human	rabbit polyclonal		Santa Cruz
ZO-1	mouse/human	rabbit polyclonal		Invitrogen GmbH, Karlsruhe, Germany
Akt-1	mouse/human	rabbit monoclonal	C73H10	New England Biolabs GmbH, Frankfurt, Germany
phospho-Akt (Ser 473)	mouse/human	rabbit polyclonal		New England Biolabs
phospho-GSK-3β (Ser 9)	mouse/human	rabbit polyclonal		New England Biolabs

### CD97 Tg and CD97 Ko Mice

A villin-CD97 expression construct was generated by placing a 9 kb regulatory region of the mouse villin gene [12] upstream of a CD97 cDNA. Mouse CD97 cDNA was amplified from a pcDNA3.1/Zeo(+)-CD97(EGF1,2,3,4) construct [13] using primers mCD97-s1/mCD97-r1 and subcloned via *Bsi*WI and *Mlu*I restriction sites into the pBS-KS-Villin-MES-SV40-polyA plasmid, kindly provided by Dr. S. Robine (Institute Curie, Paris, France). Tg mice were generated by pronuclear microinjection of the purified expression construct into fertilized oocytes of C57BL/6J mice (Harlan Winckelmann GmbH, Borchen, Germany). To establish Tg lines, founders were mated to wild-type (WT) C57BL/6J mice. WT littermates were used as controls in all experiments.

The CD97 Ko mouse has been generated recently [14] and was back-crossed to C57BL/6J mice for eight generations. All mice were housed under specific pathogen-free conditions at the Medical Experimental Center of the University of Leipzig.

### PCR

Genomic DNA was isolated from tail biopsies. Primers for amplification of the transgene were villin-s1/mCD97-r2, generating a 2100 bp PCR product. The copy number was determined in three mice of each line by quantitative real-time PCR using the primers villin-s1/mCD97-r3 and a matching external standard described previously [14].

RNA was isolated with the Qiagen total RNA isolation kits for normal or fibrous tissue (Qiagen GmbH, Hilden, Germany). cDNA was synthesized from 1 µg RNA in a 20 µl standard reaction mixture containing 200 U Superscript II RNaseH-reverse transcriptase (Invitrogen GmbH, Karlsruhe, Germany). Amplification was carried out on a Rotorgene real-time machine (Corbett Research, Mortlake, Australia) in a total volume of 20 µl containing 0.5 µM of each primer, 10 µl Power SYBR® Green PCR kit (Applied Biosystems, Darmstad, Germany), and 1 µl of DNA. The PCR primers were mCD97-s2/mCD97-r4 and mGAPDH-s1/mGAPDH-r1.

### CD97 Expression Analysis

The cleaned intestinal mucosa was removed by scratching with a glass slide. To separate single epithelial cells, the mucosa was treated for 30 s with Bashing Beads (ZymoResearch, Orange, CA) and filtered through a 40 µm gaze. Splenocytes were isolated by Ficoll-density gradient centrifugation. Cells were labeled for CD97 with the biotinylated 1B2 antibody in combination with streptavidin-phycoerythrin (Invitrogen) and analyzed on a FACS Star (BD Biosciences, Erembodegem, Belgium).

Immunohistology for murine CD97 was performed as describe [14].

### Intestinal Morphology

Cryostat sections were stained with hematoxylin-eosin (HE). The thickness of all intestinal layers, the length of the villi and the depth of the crypts were measured under a light microscope using the AxioVision program 4.5 (Carl Zeiss MicroImaging GmbH, Jena, Germany).

### AOM/DSS and DSS Models

In the AOM/DSS model 8–10 week old Tg and WT mice (n = 16/group) of both sexes were injected i.p. with 10 mg AOM (Sigma-Aldrich, Taufkirchen, Germany)/kg body weight. After one week, 2.5% DSS (MP Biomedicals, Eschwege, Germany) was added to the drinking water for 5 days, followed by 2 weeks of ordinary tap water. This cycle was repeated three times. Control mice (n = 6/group) were given water without DSS. Mice were killed 8 weeks after termination of the last cycle.

In the DSS model 8–10 week old mice (n = 8/group/time point of termination) of both sexes received 2.5% DSS in the drinking water for 5 days, followed by ordinary tap water. Mice were killed 4, 7, or 27 days after initiation of the experiment.

Our recently generated CD97 Ko mice [14] were not examined in the colitis models. Neutrophilic granulocytes that influence strongly the extent and severity of colitis strongly express CD97 [14]. Thus, CD97 Ko mice would mainly reflect the influence of CD97 on leukocytic infiltration in DSS-induced injury.

In the both models, clinical evaluation included daily monitoring of body weight, fecal blood, and diarrhea. Each parameter was scored in the disease activity index on a scale of 0–4, with 4 correlating with the most severe clinical disease [15]. After killing the mice, colon length and weight were recorded to determine the extent of shortening and intestinal edema. In addition, the weight of the spleen and mesenteric lymph nodes was determined. In the simple DSS model, gut tissue was collected for histological analysis and measurement of transepithelial resistance.

For microscopic evaluation, colonic tissue specimens were snap-frozen in liquid nitrogen and stained with hematoxylin-eosin (HE). Severity, extent of inflammation, and crypt damage were judged together with the percentage of involvement in the colon as described [16]. The maximum histological score was 40. Granulocytes were stained for myeloperoxidase by standard immunohistology.

### Whole Colon Culture and ELISA

100 mg of distal colon was cut into small pieces and cultured in 12-well culture plates with 2 ml serum-free RPMI. A high concentration of penicillin/streptomycin was supplemented to prevent bacteria growth. After incubation at 37°C for 24 h, supernatants were collected and examined for chemokine production with ELISA kits (R&D Systems GmbH, Wiesbaden, Germany).

### Electron Microscopy

Mice were analyzed at the age of 3 months. Samples of the small and large bowel (n = 3 of each mouse line) of about 1 mm^3^ were rapidly collected and fixed in cold buffered Karnovsky's solution/2% glutardialdehyde/2% paraformaldehyde (pH 7.4) for 2 h. Samples were contrasted with osmiumtetroxide and phosphotungstic acid and embedded after dehydration in Durcupan (Fluka Chemie AG, Buchs, Switzerland). Ultrathin sections were contrasted with uranyl acetate and lead citrate solution. Representative electron micrographs were captured using an EM 900 (Carl Zeiss Meditec, Oberkochen, Germany).

### Ussing Chamber Studies

Distal colonic tissue specimens were mounted in modified Ussing chambers and transepithelial resistance was recorded [17].

### Laser-Scanning Microscopy

Caco-2, SW-480, Widr, DLD-1, and Hek293 cells were obtained from The American Type Culture Collection (ATCC, Manassas, USA). HT-29/B6 cells were cultured as described [18]. CD97 was co-localized in the cell lines and in the mouse intestine with antibodies to junctional proteins by laser-scanning microscopy [14]. To examine the role of F-actin, cells were treated with 0.2 µM cytochalasin D (Sigma), an inhibitor of actin polymerization, for 30 min prior to staining. F-actin was labeled with AlexaFluor 564-coupled phalloidin (Invitrogen GmbH, Karlsruhe, Germany).

### Human Colon Specimens

Paraffin sections of inflamed colonic specimens from patients with ulcerative colitis with mild to moderate macroscopic disease activity (n = 5) were stained with a CD97 α-chain-specific polyclonal antibody (Imgenex, San Diego, CA) by immunohistology after antigen retrieval (citrate buffer, pH 6.0). Control specimens were taken from patients undergoing resection of sigmoidal or rectal cancer (n = 5).

### Immunochemical Analyses

Western blotting was performed as described [14]. Cells or tissues were lysed in M-PER or T-PER lysis buffer containing Halt™ protease inhibitor cocktail (Thermo Fisher Scientific, Bonn, Germany). The ProteoJET™ membrane protein and cytoplasmic/nuclear protein extraction kits (Fermentas GmbH, St. Leon-Rot, Germany) were used to isolate membrane, cytoplasmic and nuclear proteins.

Custom Kinetworks KCPS multiimmunoblotting analyses were performed using 300 µg of colonic lysate proteins of CD97 Ko, WT and Tg1 mice as described [19].

### Statistical Analysis

Where applicable, data are expressed as mean ± SEM. Statistical analysis was performed using the Student's t-test. p<0.05 was considered significant.

## Results

### CD97 Tg Mice Constitutively Overexpress CD97 in Intestinal Epithelial Cells

**Figure 1:**
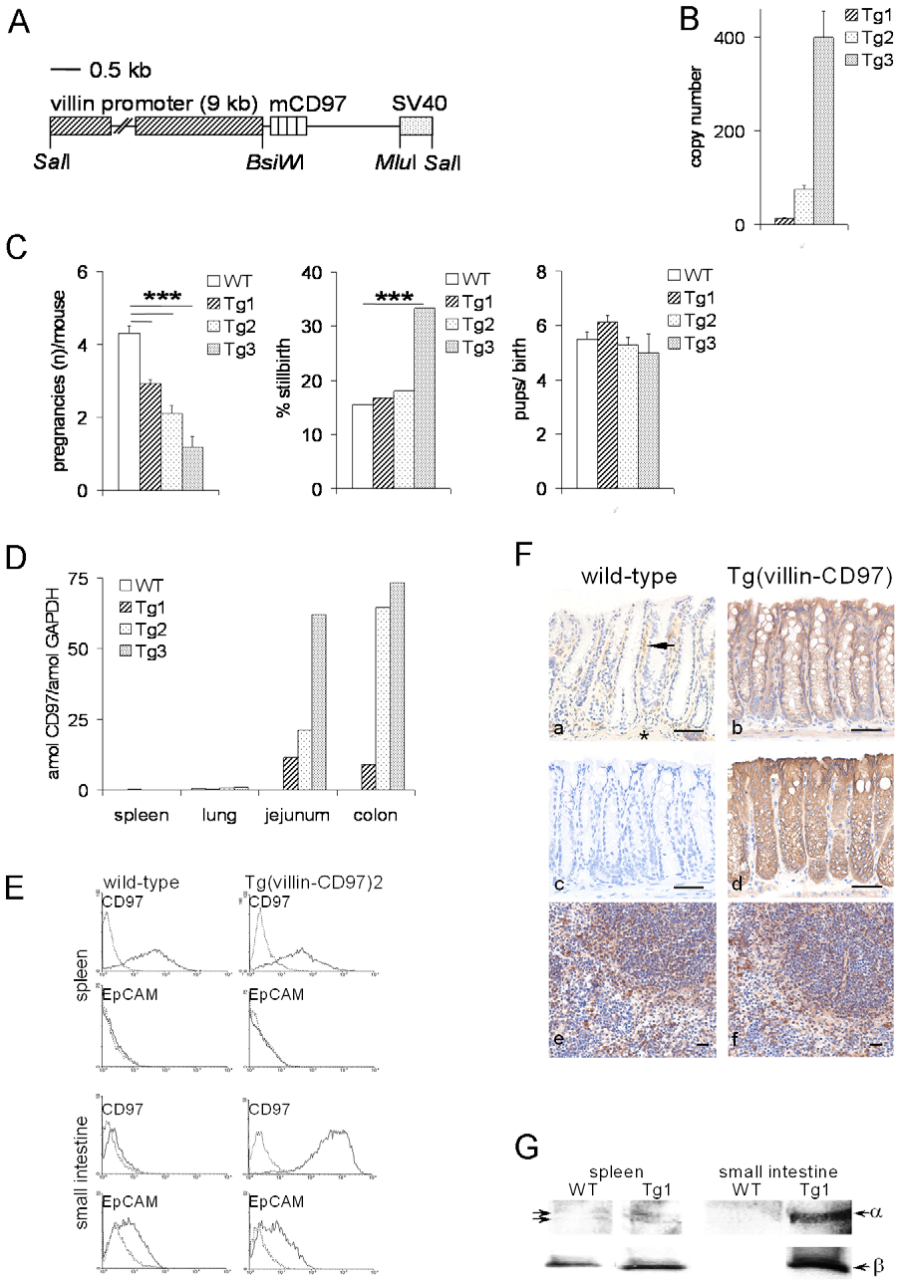
Generation of CD97 Tg mice that overexpress CD97 in intestinal cells. A Schematic representation of the pBS-KS-Villin-CD97 construct showing the villin promoter region (hatched box), the mouse CD97 cDNA containing EGF-like domains 1 to 4, and the SV40 polyadenylation sequence (dotted box). B Three CD97 Tg lines with different CD97 cDNA copy numbers were established (n = 3 mice/line, mean ± SEM). C CD97 Tg mice showed impaired reproductive performance. The number of births per mating pair correlated inversely with the CD97 cDNA copy number integrated (n = 6 mating pairs over 4 months). The percentage of stillbirths dramatically increased in the Tg3 line. The number of pups per birth was comparable between Tg and WT mice. (***, p<0.001). D Quantitative RT-PCR of tissues from WT and Tg mice. Tissues with a high percentage of villin-positive cells as the small and large intestine expressed high levels of CD97 mRNA in Tg mice. E Flow-cytometric analysis revealed comparable levels of CD97 expression in splenic lymphocytes of WT and Tg mice. In contrast, enterocytes were CD97 negative in WT but strongly CD97 positive in Tg2 mice. F CD97 immunostaining (c = negative control) of tissues of WT (a, c, e) and Tg (b, d, f) mice. In WT colon (a), cells of the lamina propria mucosae (arrow) were stained whereas in the colon of Tg1 (b) and Tg2 (d) mice, epithelial cells were CD97 positive. The muscular coat (asterisk) slightly stained for CD97. In the spleen (e, f), leukocytes equally expressed CD97 in WT and Tg mice. Scale bar = 50 µm. G Western blot analysis of CD97 with α- and β-chain-specific antibodies revealed strong signals in the small intestine of Tg1 but not WT mice. In the spleen, CD97 was detected in WT and Tg mice. Two bands at 80 and 85 kDa, derived from the α-chain-specific antibody, probably present alternatively spliced CD97 isoforms.

To study the consequences of constitutive expression of CD97 in intestinal epithelial cells, we generated Tg(villin-CD97) mice expressing full-length CD97 with all four EGF-like domains under control of the villin promotor [12] (fig. 1a). Ten independent founders were identified that had integrated the CD97 transgene. By mating with WT mice, three lines were established, designated CD97 Tg1, CD97 Tg2, and CD97 Tg3 (in short, Tg1 to Tg3). The copy number of the CD97 transgene in the genome of these lines was calculated using real-time PCR. Tg1 had integrated approximately 10–15 copies, Tg2 70–80 copies, and Tg3 nearly 400 copies (fig. 1b).

Monitoring of reproductive performance revealed an impaired fitness in the Tg mice that correlated with the CD97 cDNA copy number integrated (fig. 1c) and that presumably was due to overexpression of CD97 in villin-positive cells in the female and male reproductive tracts [20]. Tg3 was difficult to breed and could not be characterized in detail in this study.

Expression of CD97 in the small and large intestine is generally low in WT mice as compared to tissues like spleen and lung [14]. Transcription of the villin-driven transgene in the intestines resulted in a copy-number dependent increase in CD97 mRNA in the Tg mice up to 150 times-fold higher than that observed in WT mice (fig. 1d). In contrast, no change in CD97 mRNA levels was observed in spleen and lung. At the protein level, isolated intestinal cells of the small (fig. 1e) and large bowel were strongly CD97 positive in Tg but negative in WT mice. Moreover, immunohistology revealed strong CD97 expression in the epithelial layer of the small and large intestine, while in WT animals these cells were CD97 negative (fig. 1f). CD97 staining of villin-positive cells was stronger in Tg2 mice compared to Tg1 mice, indicating that also at the protein level, CD97 expression correlated with the number of CD97 cDNA copies integrated (fig. 1f).

Western blot analysis of small bowel lysates with a CD97 α-chain-specific antibody revealed a strong signal at approximately 85 kDa only in Tg mice (fig. 1g). The band corresponded to the predicted size of full-length CD97(EGF1,2,3,4). In the spleen, two bands were visible in both WT and Tg mice, and are likely to represent CD97(EGF1,2,3,4) and the smaller isoform CD97(EGF1,2,4). Signals for the CD97 β-chain at 28 kDa were obtained in the spleen of WT and Tg mice but only in the small intestine of Tg animals.

Microscopic investigation of intestinal morphology showed no changes in Tg mice as compared to WT mice (data not shown).

### CD97 Tg Mice Are Protected against DSS-Induced Colitis

**Figure 2:**
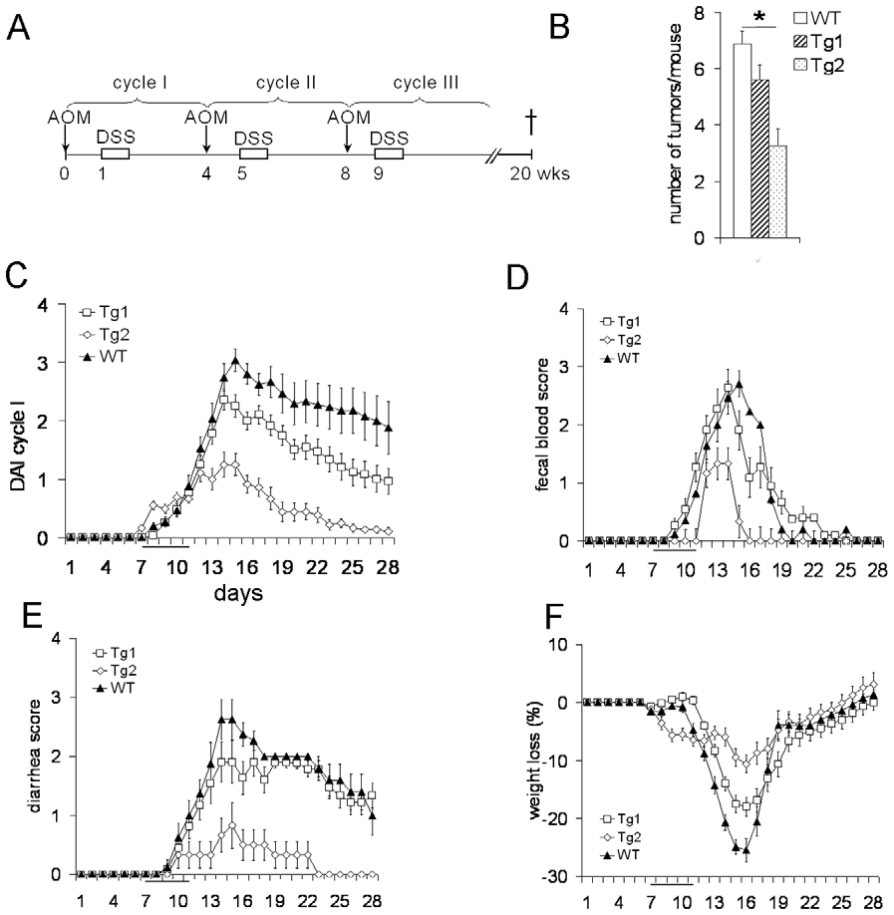
CD97 Tg mice are protected from colitis in the AOM/DSS model. A Treatment protocol: Mice underwent three cycles of AOM/DSS treatment (n = 16/group). B Intestinal CD97 overexpression changed the number of tumors in the colon of Tg mice (*, p<0.05). C–F Disease activity index (DAI) and separate scores of this index during the first treatment cycle causing acute colitis in WT animals. Disease activity (C), fecal blood (D), diarrhea (E), and weight loss (F) were strongly reduced in Tg mice. Lowest disease activity was found in Tg2 mice.

CD97 Tg mice were examined in the AOM/DSS model for colitis-associated tumorigenesis. Three cycles of AOM/DSS were administered (fig. 2a). All AOM/DSS treated mice, irrespective of the genotype, developed colorectal cancer. Intestinal CD97 overexpression decreased the number of tumors in the colon significantly in the Tg2 line and caused a trend toward fewer tumors in Tg1 mice (fig. 2b). During the first treatment cycle, application of DSS induced severe acute colitis in WT mice as judged by the assessment of body weight, fecal blood, and diarrhea. Surprisingly, disease activity was significantly reduced in both Tg(villin-CD97) lines. Each of the single parameters scored for the disease activity index was decreased in Tg compared to WT mice, thereby displaying a correlation with CD97 cDNA copy number (fig. 2c–f).

**Figure 3:**
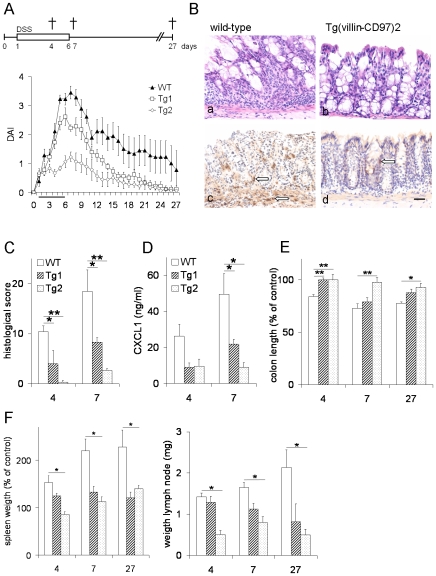
CD97 Tg mice show reduced clinical and microscopic signs of DSS-induced colitis. A Treatment protocol: Mice were killed at days 4, 7, or 27 after initiation of the experiment (n = 8/group/time point of termination). Shown here is the disease activity index (DAI) of mice that stayed in the experiment for 27 days. B Crypt damage and inflammation of the distal colon was strongly reduced in Tg2 (b, d) compared to WT mice (a, c) at day 7. Only few neutrophils (arrow) were detected in the distal colon of Tg mice. HE- (a, b) and myeloperoxidase- (c, d) stained sections; scale bar = 20 µm. C Histological examination at days 4 and 7 revealed gradually lowered colitis in Tg mice depending on the CD97 cDNA copy number integrated (*, p<0.05; **, p<0.01). D Decreased secretion of CXCL1 in whole colon cultures of Tg mice at day 7 (*, p<0.05). E Colon shortening as a hallmark of DSS-induced damage was stronger in WT compared to Tg mice at days 4, 7, and 27 (*, p<0.05; **, p<0.01). F Increase in spleen and mesenteric lymph node weight as signs of local and systemic activation of the immune system were reduced in Tg mice at days 4, 7, and 27 (*, p<0.05).

Thus, we decided to focus our investigation on the effects of CD97 overexpression on the function of the intestinal epithelium. A simple DSS model unambiguously confirmed the protective effect of CD97 overexpression in intestinal cells (fig. 3a). Histological scoring of the colon revealed more crypt damage and a markedly higher degree and extent of inflammation in DSS-treated WT mice compared to Tg mice (fig. 3b, c). Tg2 mice even had no signs of epithelial injury and inflammation at day 4 and only low activity at day 7. Quantitation of myeloperoxidase-positive cells, infiltrating into the distal colon, showed higher numbers of neutrophils in WT compared to Tg mice (fig. 3b). Epithelial damage and inflammation was mainly restricted to the distal part of the colon in all mice. Inflammatory cytokines as CXCL1 (KC), CXCL2 (MIP-2) and CCL2 (MCP-1) were clearly induced to much higher amounts in whole colon cultures of WT compared to Tg mice as exemplarily shown here for CXCL1 (fig. 3d). Colon shortening as a hallmark of DSS-induced damage was reduced in Tg compared to WT mice (fig. 3e). Finally, local and systemic activation of the immune system, illustrated by enlargement of the spleen and mesenteric lymph nodes, was markedly reduced in Tg mice (fig. 3f).

### Anchoring Cell Junctions Are Strengthened in CD97 Tg but Weakened in CD97 Ko Mice

**Figure 4:**
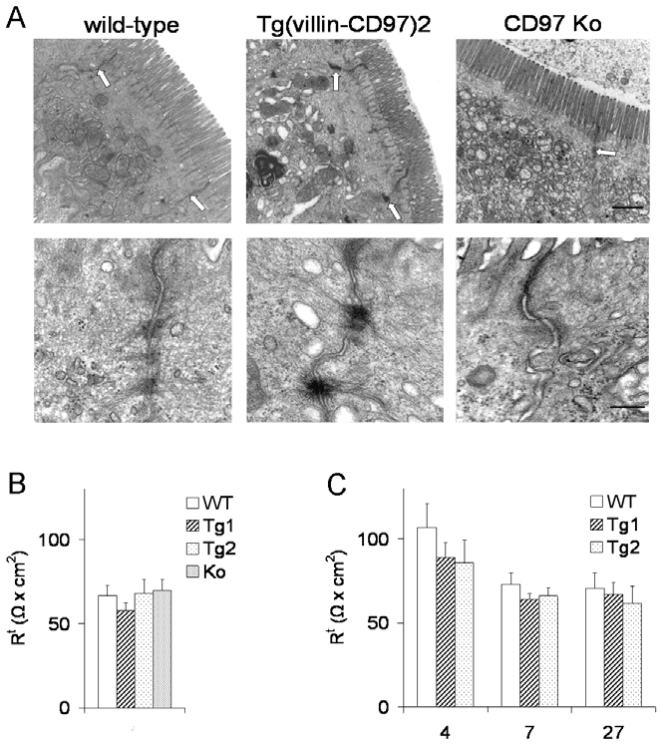
Overexpression of CD97 strengthens apicolateral cell-cell contacts between enterocytes. A Examination by electron microscopy revealed a correlation between CD97 expression and the formation of cell-cell junctions (arrows). In CD97 Tg mice especially the cytoplasmic sites of the contacts were condensed, and microfilaments were pronounced. Upper images, scale bar = 1 µm; lower images, scale bar = 0.25 µm. B Transepithelial resistance in the distal colon was comparable between untreated WT, Tg, and Ko mice (n = 4). C Transepithelial resistance remained comparable between WT and Tg mice after 4, 7, and 27 days of DSS-treatment (n = 4).

As morphology of the intestinal epithelium in CD97 Tg mice was normal at the microscopic level, we tested the possibility that CD97 increases the stability of lateral cell-cell contacts by ultrastructural analysis. Enterocytes of Tg mice appeared to be well polarized with apical membranes bearing characteristic microvilli (fig. 4a). Examining cell junctions, including the luminally localized tight junctions and the more basally localized adherens junctions, revealed that the latter anchoring contacts were strengthened in Tg mice (fig. 4a). Especially the cytoplasmic site of the junctions, characterized by a plate-like densification or plaque into which microfilaments of the cytoskeleton feed, was condensed. Here, microfilaments were extremely prominent and appeared to be continuous with the bundles of filaments that encircle the cytoplasmic surface of the contact region, building the adhesion belt of the enterocytes. In contrast, in CD97-deficient mice, adherens junctions were weakened. Here, the bundles of filaments nearly disappeared whereas tight junctions seemed to be well arranged (fig. 4a).

To examine whether CD97 affected transepithelial resistance, which crucially depends on tight but not adherens junctions, Ussing chamber experiments were performed. Transepithelial resistance was comparable in the distal colon and ileum (data not shown) of untreated WT, Tg, and Ko mice (fig. 4b). Also after DSS treatment, no differences in transepithelial resistance were observed between Tg and WT mice (fig. 4c).

### CD97 Co-Localizes with Adherens Junction Proteins

**Figure 5:**
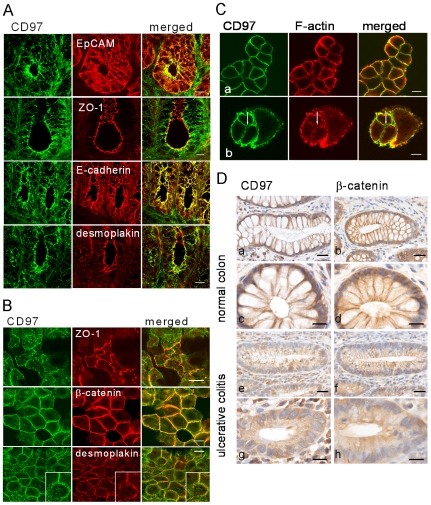
CD97 is localized in adherens junctions. A CD97 (green) was concentrated at the apicolateral region of colonic enterocytes of Tg mice as shown by doublestaining with the epithelial cell marker EpCAM (red). CD97 co-localized with the adherens junction receptor E-cadherin. In contrast, ZO-1 and desmoplakin as marker for tight junctions and desmosomes, respectively, showed only partial co-localization with CD97. Scale bar = 10 µm. B In polarized HT-29/B6 human colorectal carcinoma cells, CD97 co-localized with β-catenin but not with ZO-1 or desmoplakin. Photographs for ZO-1 co-staining were taken from the most apical part of the cells. Scale bar = 10 µm. C (a) Immunostaining of HT29/B6 cells for CD97 (green) showed partial co-localization in the lateral cell-cell contacts with cortical F-actin (red). (b) F-actin disappeared after destruction by cytochalasin while CD97 remained partly in the cortical belt (arrow, cell-cell contacts). Scale bar = 10 µm. D In human colonic enterocytes, CD97 had a similar distribution as β-catenin in lateral cell contacts. In biopsies of ulcerative colitis, CD97 expression at cell-cell contacts was partly lost. Infiltrating leukocytes strongly expressed CD97. Scale bar = 20 µm (a, b, e, f) or 10 µm (c, d, g, h).

To further explore a potential link between the expression of CD97 and the formation of adherens junctions, we applied double-immunostaining in combination with laser scanning microscopy. In colon sections of Tg mice, CD97 co-localized with the adhesive receptor E-cadherin (fig. 5a) and the anchor protein β-catenin (data not shown), two key constituents of adherens junctions. In contrast, little co-localization was found with the tight junction proteins ZO-1 (fig. 5a) and occludin (data not shown) and with the desmosom protein desmoplakin.

Staining of HT29/B6 human colorectal carcinoma cells showed consistent expression of CD97 along the lateral cell membrane, where it co-localized with β-catenin but not with ZO-1 and desmoplakin (fig. 5b). Co-localization of CD97 with β-catenin was confirmed in other colorectal cell lines that highly express CD97 at the cell surface [10] such as SW-480, Caco-2, DLD-1, and Widr (data not shown).

CD97 staining indicated that the molecule might be involved in the anchoring of cadherin family adhesive receptors to the actin filaments of the cytoskeletal network. We found partial co-staining for CD97 and cortical F-actin (fig. 5c). To test whether F-actin regulates the subcellular localization of CD97, we treated HT29/B6 cells with cytochalasin D. As a consequence, F-actin became disorganized and lost its membrane localization. However, the membrane association of CD97 was preserved and only disappeared in cells with a totally disorganized cytoskeleton (fig. 5c) suggesting that CD97 is not likely to couple to F-actin by a direct physical interaction.

We continued our investigation by examining the subcellular localization of CD97 in human colonic enterocytes. Immunostaining of paraffin sections of normal colon detected CD97 at lateral cell-cell contacts akin to the distribution of β-catenin (fig. 5d). In colonic biopsies of ulcerative colitis patients, enterocytes were CD97 positive, preferentially in the cytoplasm. Staining at lateral cell-cell junctions was observed only in one out of five patients, indicating the loss of CD97 in such cell contacts in ulcerative colitis. A similar disorganization was observed for β-catenin (fig. 5d). Quantitation of CD97 in ulcerative colitis by Western blot analysis was hampered by the high expression of the molecule on accumulating inflammatory leukocytes.

### CD97 Increases Junctional β-Catenin by Akt/GSK-3β Signaling

**Figure 6:**
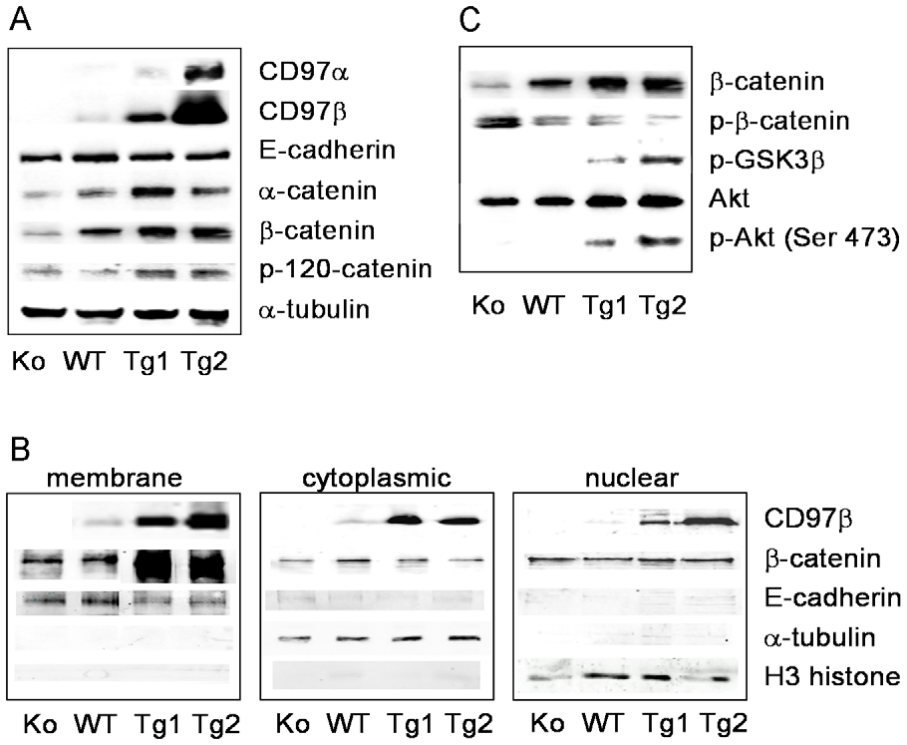
CD97 stabilizes membrane-associated β-catenin. A Western blot analysis of colonic lysates revealed that α-catenin, β-catenin, and p120 catenin were increased in Tg compared to WT mice. Lowest levels of these catenins were seen in Ko mice. Expression of E-cadherin was not affected. α-tubulin was used as a loading control. B Western blot analysis of the membrane, cytoplasmic and nuclear fraction of colonic lysates showed an increase only in membrane-associated β-catenin in Tg mice. C Phosphorylation of β-catenin, leading to its degradation, inversely correlated with the amount of CD97 in colonic lysates. Phosphorylation of the kinases GSK-3β and Akt that are involved in the regulation of β-catenin degradation was regulated by CD97 (see *Results* for details). The same lysates as in (A) were analyzed. A–C All experiments were performed in triplicate, typical runs are shown. Colonic lysates of the various mouse lines were always analyzed in one run in parallel.

We next investigated whether CD97 modulates the expression of proteins that form adherens junctions. In colonic lysates, the amount of the adhesion receptor E-cadherin was not affected in Tg and Ko mice (fig. 6a). In contrast, expression of the plaque-building proteins α-catenin, β-catenin, and p120-catenin was enhanced in Tg mice and decreased in Ko mice as compared to WT animals.

Next to the majority of β-catenin that is bound to the intracellular domain of E-cadherin, a cytoplasmic pool exists that is degraded after phosphorylation [21]. Increased levels of cytoplasmic β-catenin can accumulate and become available for nuclear translocation and binding to T-cell factor (TCF) DNA-binding proteins, resulting in gene transcription. By extracting membrane, cytoplasmic, and nuclear proteins, we could show that CD97 overexpression specifically causes accumulation of membrane-associated β-catenin while the amount of cytosolic and nuclear β-catenin was not affected and rather low (fig. 6b).

Cellular levels of β-catenin are regulated through phosphorylation by GSK-3β at Ser 33/37 leading to proteasomal degradation [21]. Interestingly, CD97 overexpression decreased phospho-β-catenin in Tg compared to WT mice. Highest levels of p-β-catenin were found in Ko mice, where probably most of the total β-catenin was phosphorylated (fig. 6c). Thus, phosphorylation of β-catenin inversely correlated with the amount of CD97, indicating that CD97 stabilizes β-catenin and increases its level in adherens junctions. Our data suggest an inactivation of GSK-3β, which occurs by phosphorylation through different kinases [22], [23]. Indeed, quantitation of phospho-GSK-3β (Ser9) revealed its increase in Tg2 compared to WT mice. Our data further suggest that GSK-3β is inactivated by Akt, known as protein kinase B (PKB), a serine/threonine kinase that is fully activated following its phosphorylation [8]. We found an upregulation of total Akt in Tg compared to WT and Ko mice combined with detectable p-Akt (Ser473) in Tg1 and Tg2 mice (fig. 6c). This result was confirmed by multiimmunoblotting analysis of colonic lysates of CD97 Ko, WT and Tg1 mice: one out of the two proteins exhibiting phosphorylation changes of greater than 300% between WT and Tg1 mice was p-Akt (Ser473; signal intensity CD97 Ko: 217, WT: 180; Tg1: 614 arbitrary units). Phosphorylation of Akt at tyrosine 308 was only detectable in Tg1 lysates (signal intensity Tg1: 78 arbitrary units).

## Discussion

We here demonstrate a novel role of the adhesion G-protein-coupled receptor CD97 in regulating intestinal epithelial cell function. By comparing CD97 Tg mice that constitutively overexpress CD97 in intestinal epithelial cells to WT animals and to mice that lack a functional *Cd97* gene, we obtained evidence that CD97 controls the structure of enterocytic adherens junctions and thereby the integrity of the intestinal barrier.

We first made these observations when subjecting CD97 Tg mice to AOM treatment combined with DSS in order to study the effect of CD97 on tumorigenesis. DSS causes destruction of the epithelial cells in the basal crypts and induce an inflammatory reaction in the colonic mucosa that acts as a promoter of colorectal carcinogenesis [24], [25]. Unexpectedly, we found a reduction of tumor numbers in Tg mice that was caused by amelioration of DSS-induced injury. Protection of CD97 Tg mice from DSS colitis was reproduced by treatment with DSS alone and involved lower clinical disease activity, less histological crypt damage, and reduced local and systemic immune reactivity.

The finding that CD97 overexpression attenuated colitis and the fact that this amelioration correlated with the CD97 cDNA copy number integrated in the Tg mice indicated that CD97 can regulate epithelial cell function. Integrity and stability of the intestinal epithelium is maintained by different types of specialized cell contacts. Immunohistochemistry revealed co-localization of CD97 with proteins of E-cadherin-based adherens junctions in murine as well as human colonic enterocytes and colorectal cell lines. In addition, electron microscopy studies showed that these basally localized cell-cell junctions were strengthened in CD97 Tg mice and weakened in CD97 Ko mice.

The formation and stabilization of adherens junctions requires the recruitment of cytosolic β-catenin to the plasma membrane and its tight association with E-cadherin [8]. We found evidence that CD97 can regulate the localization and stability of β-catenin in enterocytes. Firstly, CD97 expression levels correlated with the amount of non-phosphorylated, stable β-catenin. Secondly, the amount of p-Akt (Ser473) and p-GSK-3β was regulated consistently and reversely in Ko and Tg compared to WT mice, indicating that Akt/GSK-3β signaling is involved in the stabilization of β-catenin through CD97. Akt phosphorylates the N-terminus of GSK-3β thereby inhibiting the ability of GSK-3β to phosphorylate β-catenin [8]. Because phosphorylation of β-catenin initiates its ubiquitination and degradation in the proteasome, phosphorylation of GSK-3β may explain the accumulation of membranous non-phosphorylated β-catenin in Tg enterocytes.

In tumor cells, a different distribution of β-catenin is found. Here, accumulation of β-catenin caused by mutations in the adenomatous polyposis coli (*Apc*) gene, involved in the intracellular transport of β-catenin [26], or in the genes encoding axin and β-catenin leads to translocation of β-catenin into the nucleus, where it induces genes critical for cell transformation and cancer [27]. It becomes increasingly clear that the junctional, cytoplasmic and nuclear pools of β-catenin are closely connected [2]. In our study we identified CD97 to regulate the membrane-associated pool of β-catenin.

The observations that CD97 overexpression enhances the strength of enterocytic adherens junctions and increases random tumor cell migration [11] seem to be contradictory. We have evidence that Akt signaling is involved in both the CD97-induced strengthening of adherens junctions and in the migration of tumor cells: specific inhibitors of the phosphoinositide-3 kinase (PIK3)/Akt pathway stopped CD97-induced random tumor migration [28]. The possibility of a dual role of CD97 is supported by a recent study demonstrating Akt signaling in E-cadherin-based adherens junction formation as well as in tumor cell migration [8]. Of note is the fact that CD97 is not restricted to adherens junctions. Peripheral blood leukocytes bear the molecule evenly distributed at their surface [29], [30]. In addition, carcinomas also show intracellular cytoplasmic staining for CD97 [10], [31]. In migratory, CD97-overexpressing HT1080 cells, the molecule was located in membrane ruffles at the leading edge, co-localized with cortical F-actin [28]. Accordingly, membrane ruffles of migrating tumor fibroblasts were β-catenin positive [32]. All these data indicate that CD97 is found in different cellular pools where it might engage with additional molecular processes.

Adherens junctions crucially contribute to the organization and stabilization of a polarized intestinal epithelium and their dysregulation has been linked causally to inflammatory bowel disease [4]–. To our knowledge, the CD97 Tg mouse is the first genetic model, in which adherens junctions are strengthened and intestinal barrier function is augmented. Interestingly, a susceptibility locus for inflammatory bowel disease has been found in close proximity to the EGF-TM7 gene cluster on chromosome 19p13 [33], [34]. In addition, association of a single nucleotide polymorphism in the CD97 promotor (T64C) was associated with ulcerative colitis [35]. Although these findings need further confirmation, they may indicate a role of EGF-TM7 receptors in inflammatory bowel disease. By analyzing colonic biopsies of ulcerative colitis patients, we observed a loss of CD97 from enterocytic adherens junctions in 4 out of 5 patients. Whether this was due to the ongoing disintegration of the tissue or indicates the involvement of CD97 in the disease process remains to be shown.

In summary, by establishing CD97 Tg mice and examining them in tumor- and colitis-associated models we identified a novel, yet unknown function of the adhesion G-protein-coupled receptor CD97. CD97 regulated plaque core proteins of adherens junctions in colonic enterocytes. CD97 overexpression strengthened these cell contacts, which results in an increased epithelial integrity and decreased experimental colitis sensitivity. Moreover, for the first time key molecules of intracellular signaling via an EGF-TM7 receptor were identified. Our data suggest that CD97 induces Akt/GSK-3β signaling that results in a decrease of proteolytic digestion of β-catenin and its stabilization in adherens junctions. Our data are confirmed by the reverse results obtained in Ko mice.

## References

[1] Niessen CM, Gottardi CJ (2008). Molecular components of the adherens junction.. Biochim Biophys Acta.

[2] Kam Y, Quaranta V (2009). Cadherin-bound beta-catenin feeds into the Wnt pathway upon adherens junctions dissociation: evidence for an intersection between beta-catenin pools.. PLoS ONE.

[3] Jeanes A, Gottardi CJ, Yap AS (2008). Cadherins and cancer: how does cadherin dysfunction promote tumor progression?. Oncogene.

[4] Ivanov AI, McCall IC, Parkos CA, Nusrat A (2004). Role for actin filament turnover and a myosin II motor in cytoskeleton-driven disassembly of the epithelial apical junctional complex.. Mol Biol Cell.

[5] Zbar AP, Simopoulos C, Karayiannakis AJ (2004). Cadherins: an integral role in inflammatory bowel disease and mucosal restitution.. J Gastroenterol.

[6] Bruewer M, Samarin S, Nusrat A (2006). Inflammatory bowel disease and the apical junctional complex.. Ann N Y Acad Sci.

[7] Abe K, Takeichi M (2008). EPLIN mediates linkage of the cadherin catenin complex to F-actin and stabilizes the circumferential actin belt.. Proc Natl Acad Sci U S A.

[8] Rivard N (2009). Phosphatidylinositol 3-kinase: a key regulator in adherens junction formation and function.. Front Biosci.

[9] Yona S, Lin HH, Siu WO, Gordon S, Stacey M (2008). Adhesion-GPCRs: emerging roles for novel receptors.. Trends Biochem Sci.

[10] Steinert M, Wobus M, Boltze C, Schütz A, Wahlbuhl M (2002). Expression and regulation of CD97 in colorectal carcinoma cell lines and tumor tissues.. Am J Pathol.

[11] Galle J, Sittig D, Hanisch I, Wobus M, Wandel E (2006). Individual cell - based models of tumor - environment interactions. Multiple effects of CD97 on tumor invasion.. Am J Pathol.

[12] Pinto D, Robine S, Jaisser F, El Marjou FE, Louvard D (1999). Regulatory sequences of the mouse villin gene that efficiently drive transgenic expression in immature and differentiated epithelial cells of small and large intestines.. J Biol Chem.

[13] Hamann J, van Zeventer C, Bijl A, Molenaar C, Tesselaar K (2000). Molecular cloning and characterization of mouse CD97.. Int Immunol.

[14] Veninga H, Becker S, Hoek RM, Wobus M, Wandel E (2008). Analysis of CD97 expression and manipulation: antibody treatment but not gene targeting curtails granulocyte migration.. J Immunol.

[15] Cooper HS, Murthy SN, Shah RS, Sedergran DJ (1993). Clinicopathologic study of dextran sulfate sodium experimental murine colitis.. Lab Invest.

[16] Fitzpatrick LR, Khare V, Small JS, Koltun WA (2006). Dextran sulfate sodium-induced colitis is associated with enhanced low molecular mass polypeptide 2 (LMP2) expression and is attenuated in LMP2 knockout mice.. Dig Dis Sci.

[17] Schulzke JD, Gitter AH, Mankertz J, Spiegel S, Seidler U (2005). Epithelial transport and barrier function in occludin-deficient mice.. Biochim Biophys Acta.

[18] Kreusel KM, Fromm M, Schulzke JD, Hegel U (1991). Cl- secretion in epithelial monolayers of mucus-forming human colon cells (HT-29/B6).. Am J Physiol.

[19] Pelech S, Jelinkova L, Susor A, Zhang H, Shi X (2008). Antibody microarray analyses of signal transduction protein expression and phosphorylation during porcine oocyte maturation.. J Proteome Res.

[20] Horvat B, Osborn M, Damjanov I (1990). Expression of villin in the mouse oviduct and the seminiferous ducts.. Histochemistry.

[21] Aberle H, Bauer A, Stappert J, Kispert A, Kemler R (1997). beta-catenin is a target for the ubiquitin-proteasome pathway.. EMBO J.

[22] Yost C, Torres M, Miller JR, Huang E, Kimelman D (1996). The axis-inducing activity, stability, and subcellular distribution of beta-catenin is regulated in Xenopus embryos by glycogen synthase kinase 3.. Genes Dev.

[23] Srivastava AK, Pandey SK (1998). Potential mechanism(s) involved in the regulation of glycogen synthesis by insulin.. Mol Cell Biochem.

[24] Kitajima S, Takuma S, Morimoto M (1999). Changes in colonic mucosal permeability in mouse colitis induced with dextran sulfate sodium.. Exp Anim.

[25] Neufert C, Becker C, Neurath MF (2007). An inducible mouse model of colon carcinogenesis for the analysis of sporadic and inflammation-driven tumor progression.. Nat Protoc.

[26] Faux MC, Ross JL, Meeker C, Johns T, Ji H (2004). Restoration of full-length adenomatous polyposis coli (APC) protein in a colon cancer cell line enhances cell adhesion.. J Cell Sci.

[27] Wong NA, Pignatelli M (2002). Beta-catenin–a linchpin in colorectal carcinogenesis?. Am J Pathol.

[28] Aust G, Sittig D, Wandel E, Wobus M, Galle J (2007). Der Zelloberflächenrezeptor CD97 stimuliert die Migration von Tumorzellen durch Interaktion mit dem Aktin-Zytoskelett.. Chirurgisches Forum.

[29] Eichler W, Aust G, Hamann D (1994). Characterization of the early activation-dependent antigen on lymphocytes defined by the monoclonal antibody BL-Ac(F2).. Scand J Immunol.

[30] Leemans JC, te Velde AA, Florquin S, Bennink RJ, de Bruin K (2004). The epidermal growth factor-seven transmembrane (EGF-TM7) receptor CD97 is required for neutrophil migration and host defense.. J Immunol.

[31] Aust G, Eichler W, Laue S, Lehmann I, Heldin N-E (1997). CD97: A dedifferentiation marker in human thyroid carcinomas.. Cancer Res.

[32] Johnson M, Sharma M, Jamieson C, Henderson JM, Mok MT (2009). Regulation of beta-catenin trafficking to the membrane in living cells.. Cell Signal.

[33] Rioux JD, Silverberg MS, Daly MJ, Steinhart AH, McLeod RS (2000). Genomewide search in Canadian families with inflammatory bowel disease reveals two novel susceptibility loci.. Am J Hum Genet.

[34] Mathew CG, Lewis CM (2004). Genetics of inflammatory bowel disease: progress and prospects.. Hum Mol Genet.

[35] Zhang L, Yang X, Cummings F, McGovern D, Jewell D (2004). Polymorphisms in the genes of epidermal growth factor module-containing mucin-like hormone recpetor 1, 2,3 (EMR1, 2, 3) and CD97 in association with inflammatory bowel disease (IBD).. Human Genome Meeting 2004 (HGM 2004), abstract.

[36] Kwakkenbos MJ, van Lier RA, Hamann J (2002). Characterization of EGF-TM7 family members by novel monoclonal antibodies. In: Mason D, editors. Leucocyte Typing VII.. White Cell Differentiation Antigens.

